# Network Pharmacological Analysis of a New Herbal Combination Targeting Hyperlipidemia and Efficacy Validation In Vitro

**DOI:** 10.3390/cimb45020086

**Published:** 2023-02-04

**Authors:** Tae-Hyoung Kim, Ga-Ram Yu, Hyuck Kim, Jai-Eun Kim, Dong-Woo Lim, Won-Hwan Park

**Affiliations:** 1 Department of Diagnostics, College of Korean Medicine, Dongguk University, Goyang-si 10326, Republic of Korea; 2 Institute of Korean Medicine, Dongguk University, Goyang-si 10326, Republic of Korea; 3 Department of Pathology, College of Korean Medicine, Dongguk University, Goyang-si 10326, Republic of Korea

**Keywords:** network pharmacology, GO enrichment analysis, key target validation, hyperlipidemia, hepatic steatosis, herbal combination, combinational effect, *Arum ternata*, *Poria cocos*, *Zingiber officinale*

## Abstract

The network pharmacology (NP) approach is a valuable novel methodology for understanding the complex pharmacological mechanisms of medicinal herbs. In addition, various in silico analysis techniques combined with the NP can improve the understanding of various issues used in natural product research. This study assessed the therapeutic effects of *Arum ternata* (AT), *Poria cocos* (PC), and *Zingiber officinale* (ZO) on hyperlipidemia after network pharmacologic analysis. A protein–protein interaction (PPI) network of forty-one key targets was analyzed to discover core functional clusters of the herbal compounds. The Kyoto Encyclopedia of Genes and Genomes (KEGG) pathway and gene ontology (GO) term enrichment analysis identified significant categories of hypolipidemic mechanisms. The STITCH database indicated a high connection with several statin drugs, deduced by the similarity in targets. AT, PC, and ZO regulated the genes related to the energy metabolism and lipogenesis in HepG2 cells loaded with free fatty acids (FFAs). Furthermore, the mixture of three herbs had a combinational effect. The herbal combination exerted superior efficacy compared to a single herb, particularly in regulating acetyl-CoA carboxylase (ACC) and carnitine palmitoyltransferase 1 (CPT-1). In conclusion, the network pharmacologic approach was used to assess potential targets of the herbal combination for treatment. Experimental data from FFA-induced HepG2 cells suggested that the combination of AT, PC, and ZO might attenuate hyperlipidemia and its associated hepatic steatosis.

## 1. Introduction

Hyperlipidemia is a status of an elevated lipid profile in the blood due to lipid metabolic disorders [1]. The WHO announced that hyperlipidemia and high blood pressure, along with alcohol consumption and smoking, are the major causes of fatality in recent years [2]. Hyperlipidemia is generally treated with dietary intervention and hypolipidemic agents by a clinical practitioner according to the lipid profiles of the patient [3]. The clinical features of hyperlipidemia are elevated lipids in the bloodstream, including fat, fatty acids, cholesterol, phospholipids, and triglycerides [4]. The reported environmental factors of hyperlipidemia were obesity, excessive alcohol intake, BMI, and genetic factors, including LDL (low-density lipoprotein), apoA-I, apoE, and microsomal triglyceride transfer protein [5,6]. Dietary patterns fundamentally impact increased LDL cholesterol levels [7]. Elevated LDL cholesterol is a significant clinical marker of patients with atherosclerosis or several cardiovascular diseases because it describes disorders in lipid profiles [8]. The significant complications of hyperlipidemia include atherosclerosis, coronary artery disease, myocardial infarction, and ischemic stroke [9]. Reducing LDL cholesterol is considered the first goal in treating hyperlipidemia to lower the risk of cardiovascular disease [3]. 

The first-line medication for hyperlipidemia includes statins, whose mechanism is the inhibition of HMG-CoA reductase, which is the rate-limiting enzyme of cholesterol synthesis [9]. Studies have reported rhabdomyolysis, muscle pain, and myositis as side effects of statin drugs [10]. Increased serum statin concentration or decreased body muscle mass increases the risk of statin-associated muscle symptoms [10]. Statin treatment has been associated with new-onset diabetes, but the exact mechanisms are unknown [11,12]. 

The recent trend in studying mechanisms of herbal medicine adopts network pharmacology as a feasible tool for mapping the known interactions and analyzing networks between natural drugs and target genes to identify potential targets based on multi-compound and multi-target theory [13]. This approach, based on a widely existing database, can form an initial understanding of the mechanism of action of many polyherbal prescriptions [14]. One study showed that the Network Pharmacology techniques presented significant results for hyperlipidemia [15]. The characteristics of symptoms from hyperlipidemia and chronic metabolic disorders can be recognized as ‘phlegm’, which is a pathological byproduct in oriental medicine, and differentiated or treated as ‘damp-phlegm’ phenotype (or pattern) patient by practitioners [16]. Banxia Baizhu Tianma Decoction (BBTD) is a representative prescription that treats patients who have phlegm patterns with oriental medicine [17]. In addition, there are reports that BBTD attenuated high blood pressure [17]. Yijin-tang is a traditional prescription for hyperlipidemia and atherosclerosis in oriental medicine [18]. In addition, Yijin-tang improved systemic inflammation caused by obesity, which can cause hyperlipidemia, and improved insulin resistance [19]. The common medicinal herbs of these prescriptions are Pinelliae Tuber (*Arum ternata*, AT), Poria Sclerotium (*Poria cocos*, PC), and Zingiberis Rhizoma (*Zingiber officinale*, ZO). These three herbs were also listed in the top five most frequently found herbs in phlegm-eliminating herbal prescriptions, according to a recent study [20]. ZO inhibited lipid peroxidation using a high-cholesterol-fed rat model by scavenging radicals. PC was proven to be effective in a high-fat-diet-induced hyperlipidemia rat model via modulating the metabolic pathways [21,22]. In addition, AT lowered the blood triglyceride and free fatty acid levels when administered for six weeks using an obese mouse model [23]. 

Non-alcoholic fatty liver disease (NAFLD) is one of the most common metabolic liver disorders worldwide and is closely associated with metabolic syndromes, such as dyslipidemia and obesity [24]. The main characteristic of NAFLD is too much fat stored in the liver [25]. Evidence suggests that NAFLD causes dyslipidemia, such as hyperlipidemia [26]. Decreased LDLR expression on the cell surface of NAFLD may discourage the removal of LDL cholesterol from the sera [27]. Therefore, targeting NAFLD is a potential strategy for attenuating hyperlipidemia [26]. 

The phosphatidylinositol-3-kinase (PI3K)/protein kinase B (AKT) pathway is an intracellular signal transduction pathway that plays a pivotal role in glucose, lipid, and protein metabolism [28]. The level of AKT phosphorylation decreased in the muscle, liver, and visceral adipose tissues in obese patients with NAFLD [29]. In NAFLD, the phosphorylation of AMP-activated protein kinase (AMPK) decreases fatty acid and cholesterol synthesis by regulating the expression of adipogenesis genes (Acetyl-CoA carboxylase, ACC; Peroxisome proliferator-activated receptor gamma, PPARG; CCAAT Enhancer Binding Protein Alpha, CEBPA; and 3-Hydroxy-3-methylglutaryl-CoA Reductase, HMGCR), as well as increasing the expression of fatty acid oxidation and lipolysis genes, such as serine/threonine kinase 1 (CPT1) [30,31,32]. AMPK directly impacts lipid metabolism as a regulator of energy expenditure, but FFAs directly impair hepatic glucose and lipid homeostasis [33]. 

Using the latest network pharmacology technique and online pharmacological database, this study analyzed the components and target information of three herbal medicines to find targets and mechanisms that can modulate hyperlipidemia. In addition, an in vitro study was performed to verify the mechanism of these herbs estimated by network pharmacology using a fatty acid-induced hepatic steatosis model. Finally, an analysis of the efficacy and mechanism of the three medicinal herbs that can be used in the prescription of hyperlipidemia highlights their potential for treating hyperlipidemia.

## 2. Materials and Methods

### 2.1. Data Preparation

#### Data Acquisition of Herbs from the Online Database

The TCMSP (https://tcmsp-e.com accessed on 12 August 2022) [34], which is a pharmacology database of Traditional Chinese Medicine, was used to collect the compounds and targets of the herbal combination. The oral bioavailability (OB) and drug-likeness (DL) were used for screening of bioactive compounds [18]. OB represents the rate and extent of an active ingredient or active moiety that is absorbed into the blood circulation and becomes available at the site of action [35]. DL can describe the molecular properties affecting the pharmacodynamics and pharmacokinetics [36]. The threshold values of the two indices were ≥30% (OB) and ≥0.18 (DL) [37]. The results from the TCMSP database were reinforced using the OASIS database to sum the additional bioactive compounds with proper literature-based evidence for their activity (https://oasis.kiom.re.kr/accessed on 12 August 2022).

### 2.2. Hyperlipidemia-Associated Targets Prediction

Information on hyperlipidemia-associated target genes was collected from the DisGeNET (https://www.disgenet.org/, accessed on 17 August 2022) and genecards (https://www.genecards.org/, accessed on 17 August 2022) [38,39]. “Hyperlipidemia” was used for the search, and only Homo sapiens proteins were selected. The distributional results of targets and compounds from herbs were presented as Venn diagrams using bioinformatics and evolutionary genomics website (http://bioinformatics.psb.ugent.be/webtools/Venn/ (accessed on 17 August 2022)) [40]. 

### 2.3. Protein–Protein Interaction (PPI) Network Construction

A PPI network with STRING (Switzerland) was produced using a query list of target genes and exported to Cytoscape software version 3.9.1 (USA), a free software package for visualizing, modeling, and analyzing molecular and genetic interaction networks (confidence score = 0.400) [41]. The total PPI network was clustered functionally using MCODE (USA) and analyzed further with ClueGO (USA) and CluePedia (USA), which are Cytoscape plugins (USA) [41,42,43]. The STRING database aims to provide a critical assessment and integration of protein–protein interactions, including direct interactions [44]. A protein–chemical network was then screened using the STITCH database version 5.0 (USA) by submitting a target gene list [45].

### 2.4. Gene Ontology (GO) Terms and KEGG Pathway Enrichment Analysis

The enrichment of GO terms of BP was analyzed in the DAVID database (USA). Kyoto Encyclopedia of Genes and Genomes (KEGG) signaling-pathway enrichment analysis was performed using the Database for Annotation, Visualization, and Integrated Discovery (DAVID) v.6.8 (https://david.ncifcrf.gov/, accessed on 29 August 2022) [46]. A list of target genes was submitted, and the gene identifier was set to ‘official gene symbol’. 

The false discovery rate (FDR) was used as a statistical test method of the enrichment analysis in the DAVID database, which is based on fisher’s exact test [47]. The data of relative gene ratio, adjusted *p*-value, and gene counts of each GO term and KEGG were processed and presented as bubble plots using the R package (ggplot2) and public script in R studio (USA) [48].

### 2.5. Chemicals and Antibodies

Dulbecco’s Modified Eagle’s Medium (DMEM) was purchased from Hyclone (Logan, UT, USA), and fetal bovine serum (FBS) and penicillin/streptomycin solution were purchased from Invitrogen (Carlsbad, CA, USA). The EZ-Cytox assay kit, obtained from Daeil Lab Service (Chungcheongkuk-do, South Korea), was used to measure cell viability. The phosphorylation-form or non-phosphorylation-form primary antibodies of ACC (Acetyl-CoA carboxylase), AMPK (AMP-activated protein kinase), AKT (Protein kinase B), CPT-1 (Carnitine palmitoyltransferase-1) were purchased from Cell signaling Technology (Berkeley, CA, USA), and β-actin was obtained from Santa Cruz Biotechnology (Santa Cruz, CA, USA), which also supplied secondary antibodies. The oligonucleotide primers for real-time qPCR were produced by Macrogen (Seoul, South Korea).

### 2.6. Preparation of Samples

Dried herbs of *A. ternata* (AT), *P. cocos* (PC), and *Z. officinale* (ZO) were purchased from Herbmaul (Chungcheongbuk-do, South Korea). To prepare the extract, dried herbs (100 g) were ground to a powder and extracted with 500 mL in distilled water at 100 °C for 15 min. In addition, the mixed sample was blended with equal weights of AT, PC, and ZO (33 g each). The hot water extracts were filtered twice through 8 μm-pore-size Whatman filter paper, concentrated by rotary evaporation (Buchi, Flawil, Switzerland), and freeze-dried to obtain lyophilized extracts of AT, PC, and ZO. These were then eluted with DPBS and filtered through a 0.22 μm syringe filter before cell treatment.

### 2.7. Cell Culture and Treatment

HepG2 cells (a human hepatocellular carcinoma cell line) were purchased from the Korean Cell Bank (no. 88065, Seoul, South Korea) and cultured in DMEM supplemented with a 1% penicillin/streptomycin and 10% FBS at 37 °C in humidified 5% CO_2_ environment. To evaluate anti-intracellular lipid accumulation effects, the FFAs (oleic acid and palmitic acid, 2:1, v/v, respectively) were dissolved in DMSO at 1 mM concentrations. The HepG2 cells were serum-starved for 12 h, cultured with serum-free DMEM containing 1% bovine serum albumin (BSA), and exposed to 1 mM FFAs [49]. Each sample was co-treated during incubation in FFA-BSA complex media for 24–48 h for further analysis. 

### 2.8. Cell Viability Assay

The cell viability of HepG2 cells was determined using the EZ-Cytox cell viability assay kit (Seoul, South Korea) according to the manufacturer’s instructions with slight modifications [50]. Briefly, HepG2 cells were plated at a density of 4 × 10^4^ cells/well in 96-well plates. After 24 h incubation, the medium was changed to FBS-free DMEM containing a serially diluted sample (0–50 µg/mL), treated with different concentrations, and incubated at 37 °C in a humidified containing 5% CO_2_ for 24 h. Then, 10 µL of EZ-Cytox reagent was added to each well, and cells were incubated for 1 h. Optical densities (ODs) were measured at 450 nm using a microplate reader (Versamax, Molecular Devices, CA, USA).

### 2.9. Western Blot Analysis

The protein levels related to hepatic steatosis were measured by Western blot. The whole protein was isolated using a RIPA buffer (Thermo Fisher Scientific, Rockford, IL, USA) containing a protease and phosphatase inhibitor cocktail (Gendepot, Barker, TX, USA) after the cells were washed with Dulbecco’s phosphate-buffered saline (DPBS). The protein concentrations were determined using a BCA kit (Thermo Fisher Scientific, Rockford, IL, USA). Equal amounts of protein sample (40 µg/mL) were mixed with the 5× Lane Marker Reducing sample buffer (BioRad, Hercules, CA, USA) and denatured at 95 °C for 10 min. The protein lysates were loaded into 7.5% SDS-PAGE gels, electrophoresed, and transferred to PVDF membranes activated by methanol at 100 V for 60 min using an electrophoretic transfer cell (Bio-rad, Hercules, CA, USA). The membranes were blocked with 5% BSA in TBS/T (TBS containing 0.1% Tween 20) for 2 h at room temperature. The blots were incubated with primary antibodies (diluted at 1:1500 in TBS/T containing 3% BSA) overnight at 4 ℃ with gentle shaking. After washing with TBS/T, the membranes were incubated with secondary antibodies (diluted at 1:3000 in TBS/T containing 1% BSA) at room temperature for 3 h. The membrane was detected using a Western blot imaging system (Fusion Solo chemiluminescence system, Vilber Lourmat, Collegien, France), and proteins were visualized using a Super Signal West Pico ECL buffer (Thermo Fisher Scientific, Rockford, IL, USA) [51].

### 2.10. Quantitative Real-Time Polymerase Chain Reaction

The expression levels related to hepatic steatosis were determined using a quantitative real-time polymerase chain reaction (qPCR). The total RNA was isolated from HepG2 cells using a TRIzol reagent (Thermo Fisher Scientific, Rockford, IL, USA). According to the manufacturer’s instructions. Briefly, reverse transcription was performed using AccuPower RT PreMix (Bioneer, Daejeon, Republic of Korea) and oligo deoxythymine (dt) 18 primers (Invitrogen, Carlsbad, CA, USA). Primer-specific binding cDNA was amplified on a Light Cycler 480 PCR system (Roche, Basel, Switzerland) using 10 µL of SYBR green fluorescence dye Mastermix (Roche, Basel, Switzerland), 8 µL of ultrapure water, 1 pmol/µL of primer, and 1 µL of template cDNA. At least 45 amplification cycles consisting of denaturation at 95 °C for 10 min, annealing at 55–58 °C for 15 s, and extension at 72 °C for 15 s. The following primers were used: CEBPA-forward, 5′-GCGCAAGAGCCGAGATAAAG-3′, reverse, 5′-CACGGCTCAGCTGTTCCA-3′; PPARG-forward, 5′-ATGCCAAAAATATCCCTGGTTTC-3′, reverse, 5′-GGAGGCCAGCATGGTGTAGA-3′; ACC-forward, 5′-TGGCGTCCGCTCTGTGATA-3′, reverse, 5′-CATGGCGACTTCTGGGTTG-3′; and HMGCR-forward, 5′-TGATTGACCTTTCCAGAGCAAG-3′, reverse, 5′-CTAAAATTGCCATTCCACGAGC-3′; and β-actin-forward, 5′-GACGGCCAGGTCATCACTATTG-3′, reverse, 5′-CCACAGGATTCCATACCCAAGA-3′, used as the internal control. Ct results with a melting curve were checked using Roche LightCycler 480 software (Roche Applied Science, Carlsbad, CA, USA). The Ct values for the expression of each gene were normalized using the Ct values of β-actin gene expression [52].

### 2.11. Oil Red O Staining

The lipid accumulation was estimated by staining the intracellular lipid droplets with the Oil Red O reagent (Thermo Fisher Scientific, Rockford, IL, USA). HepG2 cells were plated at a density of 4 × 10^5^ cells/well in six-well plates. After 24 h, the cells were incubated with the samples (25, 50 µg/mL) and FFAs (1 mM) for 48 h. The cells were washed with DPBS and then fixed with 10% formalin for one hour at room temperature. After fixation, the cells were washed with 60% isopropanol and stained with a prepared working solution of Oil Red O for 15 min. The stained cells were washed three times with ultrapure water and dried. The cells were examined under an inverted microscope system with the camera (DMI 6000, Leica, Wetzlar, Germany), and the stains were re-dissolved in pure isopropanol to measure OD at 520 nm wavelength [53].

### 2.12. Statistical Analysis

The analysis was conducted using one-way ANOVA in Graph Pad Prism version 5.0 software (Graph Pad, La Jolla, CA, USA). The standard curves were constructed using Excel and PowerPoint (Microsoft, Redmond, WA, USA). The significance of the differences between the untreated controls and the FFs-treated cells, and between the FFA-treated and the sample-treated cells, were determined. The results are presented as means ± SDs, and *p*-values of <0.05 were considered significant.

## 3. Results

### 3.1. Selection of Potential Compounds from AT, PC, and ZO

**Figure 1:**
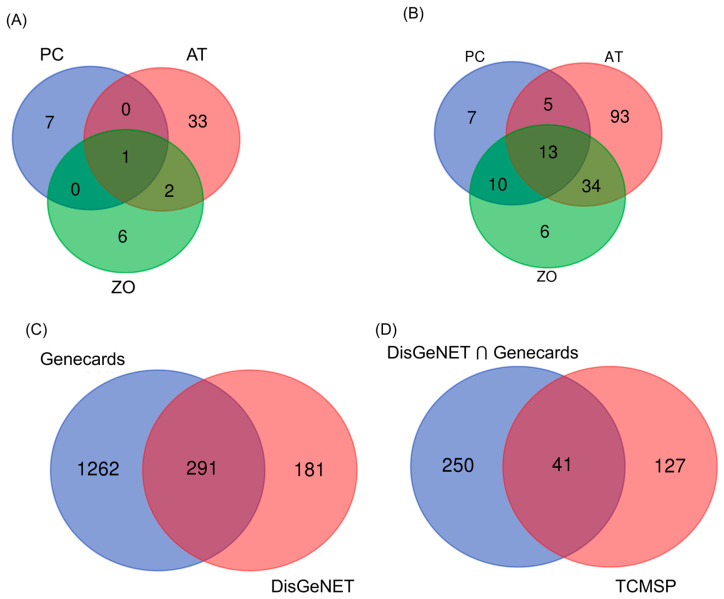
Venn diagrams of the compounds and target genes from the herbal combination. (**A**) Distribution of potential compounds from PC, AT, and ZO. (**B**) Target of potential form PC, AT, and ZO (**C**) Hyperlipidemia-related target genes from two web databases. (**D**) Dyslipidemia-related common target genes from both web databases and TCMSP.

The data of AT, PC, and ZO compounds and targets were retrieved from TCMSP and OASIS. Table 1 lists the compounds studied, and Figure 1A presents a data plot. ZO, PC, and AT have 6, 7, and 33 compounds; all three herbs commonly share one compound (palmitic acid). 

### 3.2. Target Prediction

In total, 168 and 291 targets for hyperlipidemia from disease databases were screened for three herbs from TCMSP (Figure 1B,C). As a result, 41 common targets assumed to be involved with hyperlipidemia were obtained between the target lists of the disease database and TCMSP (Figure 1D). The distributions of targets were visualized using a Venn diagram.

### 3.3. PPI Networks Construction and Analysis

**Figure 2:**
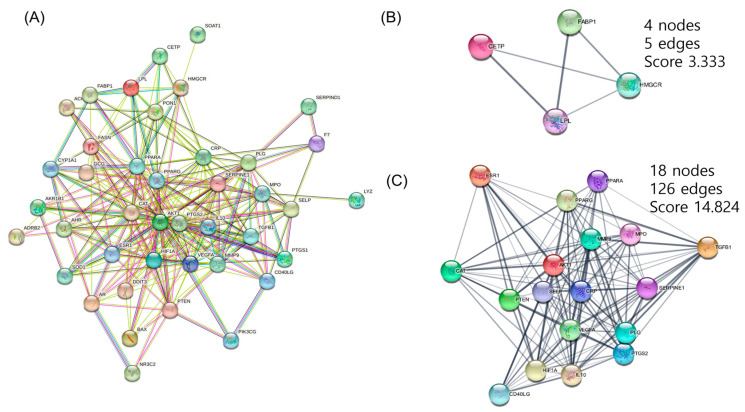
(**A**) Full protein–protein interaction network of 41 hyperlipidemia-related target genes from the herbal combination. (**B**,**C**) Functional cluster of full PPI network created from MCODE of Cytoscape.

The PPI Network was built for 41 targets expected to be significant for hyperlipidemia using the STRING Database. The full network consisted of 41 nodes and can be divided into two clusters using the MCODE of Cytoscape (Figure 2). The targets were arranged in order of importance of network parameters of degree, betweenness centrality, and stress. AKT Serine/Threonine Kinase 1 (AKT1), peroxisome proliferator-activated receptor γ (PPARγ), and prostaglandin-endoperoxide synthase 2 (PTGS2) act as important targets (Table 2).

### 3.4. CTP Visualization of Cytoscape

**Figure 3:**
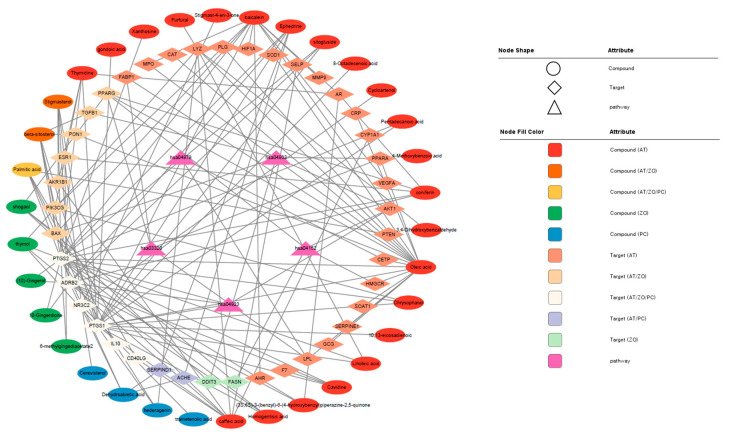
Visualization of the compounds–targets–pathways network constructed with the components of the herbal combination.

Forty-one targets were analyzed through the KEGG pathway. Based on this, the compounds–targets–pathways network was visualized in Cytoscape (Figure 3). The top five pathways expected to be effective for hyperlipidemia were identified by importing 41 targets into the DAVID database. Each node was connected to the edges of the compounds, targets, and pathways that are expected to be correlated with each other. Table 3 lists the top enriched KEGG pathways related to lipid metabolism analyzed using the target gene list. 

### 3.5. Network Analysis Using ClueGO, CluePedia

**Figure 4:**
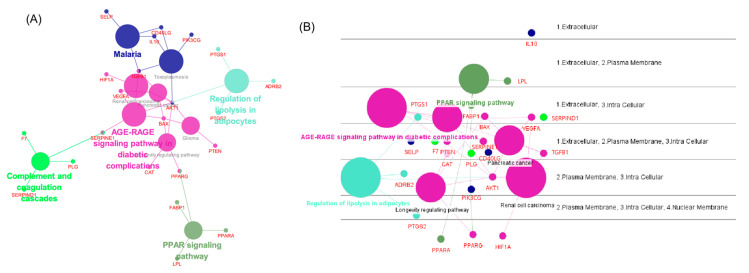
Visualization of the KEGG pathways and targets of the herbal combination analyzed by ClueGO and CluePedia. (**A**) Visualization of significantly enriched KEGG pathways and their genes (created with ClueGO) (**B**) Cerebral layout of significant KEGG pathways and their genes by their cellular compartment (created with CluePedia).

Using the full target list of the herbal combination, the network of major pathways and its component targets was visualized using ClueGO and CluePedia in Cytoscape (Figure 4). The ClueGO network could identify and visualize the interaction of pathways and their targets. As a result, the pathway of the ‘AGE-RAGE signaling pathway in diabetic complication’ interconnected two other significant pathways in lipid disorders, such as ‘regulation of lipolysis in adipocytes’ and ‘PPAR signaling pathway’, with the core target of AKT1 or PPARG (Figure 4A). In addition, CluePedia illustrated the layout of the disposition of major pathways and their component genes according to their cellular layer of localization (Figure 4B). 

### 3.6. BP and KEGG Enrichment Analysis

**Figure 5:**
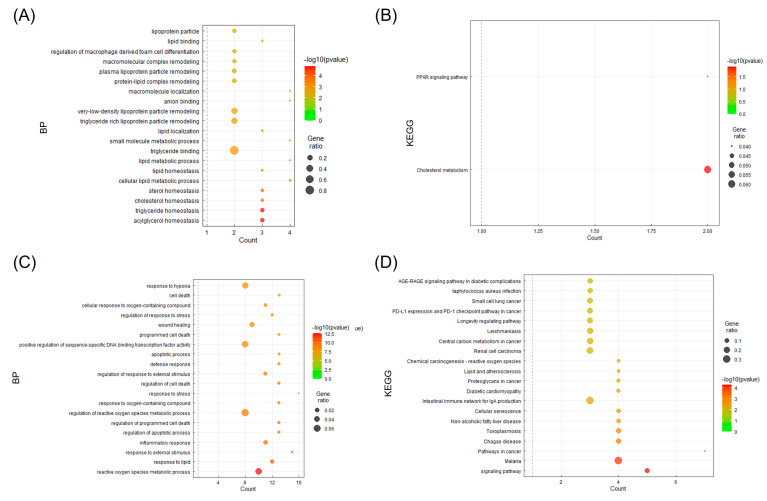
Bubble plot visualization of enrichment analysis (BP terms and KEGG pathways) from two target clusters from the herbal combination. Significant (**A**) BP terms and (**B**) KEGG pathways of target cluster 1. Significant (**C**) BP terms and (**D**) KEGG pathways of target cluster 2.

For a detailed analysis of the 41 targets, two clusters divided using Cytoscape’s MCODE app were analyzed to obtain information on the pathways of the candidate targets through the KEGG pathway analysis in the DAVID database, and each cluster was represented using the bubble plot of the GGPlot2 package (Figure 5). A and B are the results of cluster 1, confirming that they had significant results for cholesterol, triglyceride, the PPAR signaling pathway, and cholesterol metabolism. C and D are the results of cluster 2. Significant results were confirmed for lipid response, non-alcoholic fatty liver, and atherosclerotic disease.

### 3.7. Visualization of the Target–Chemical Interaction Using STITCH

**Figure 6:**
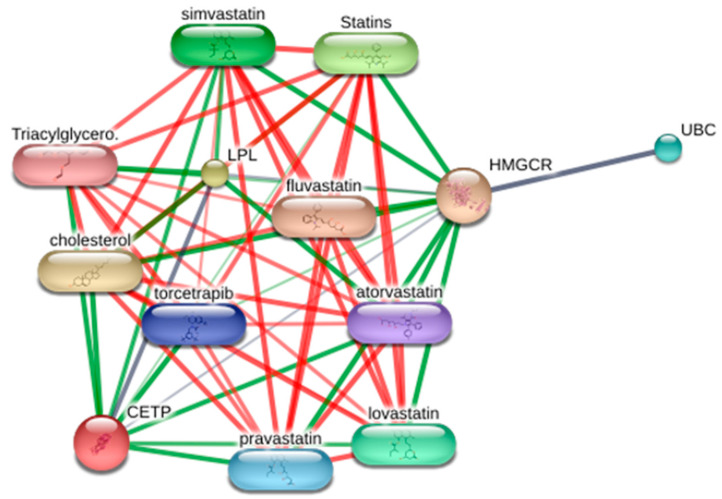
Visualization of the target–chemical interaction of targets from cluster 1 using the STITCH database.

As the enrichment analysis results from the functional cluster indicated the significant potential of targets on lipid disorders, this study investigated the chemical-target network in the STITCH database (Figure 6). As a result, the targets from cluster 1 harbor strong interactions with various currently used statins.

### 3.8. AT, PC, ZO, and Mixed Extract (MIX) Improved the Energy Metabolism-Related Proteins in the Hepatic Steatosis Model

**Figure 7:**
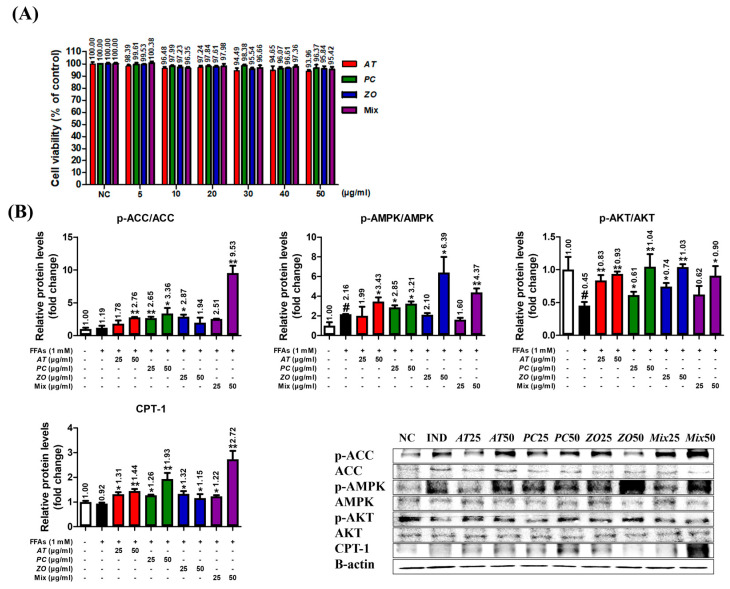
AT, PC, ZO, and MIX stimulate ACC, AMPK, and AKT phosphorylation in FFA-induced hepatic steatosis and activate CPT-1 related to fatty acid oxidation. HepG2 cells were incubated in the absence or presence of FFAs (1 mM) with AT, PC, ZO, and MIX for 24 h. (**A**) HepG2 cells were treated with various concentrations (0–50 µg/mL) of AT, PC, ZO, and MIX for 24 h. The results are presented as means ± SDs of the percentages determined by three independent experiments versus the non-treated controls. (**B**) HepG2 cells were co-treated with each sample and FFAs for 24 h. Western blot analysis shows the effect on the phosphorylation of ACC and AMPK and AKT protein expression related to the energy metabolism and lipogenesis in FFA-induced hepatic steatosis HepG2 cells. The band intensities were measured by densitometry and normalized versus the intensities of the total forms and β-actin. The results are presented as the means ± SDs of three independent experiments. # *p* < 0.05 versus FFA-treated controls, and * *p* < 0.05, ** *p* < 0.01 versus FFA-treated HepG2 cells.

The HepG2 cells did not show any significant decrease in viability at <50 µg/mL (93.96%, 96.37%, 95.84%, and 95.42% at 50 µg/mL of AT, PC, ZO, and MIX, respectively) (Figure 7A). The influence on the protein expression of ACC, AMPK, AKT, and CPT-1 was investigated in the steatosis model. At 50 µg/mL, AT and PC stimulated the phosphorylation of ACC, AMPK, and AKT and increased CPT-1 expression related to fatty acid oxidation. In addition, ZO promoted the phosphorylation of AMPK and AKT, and the expression of CPT-1. In particular, MIX strongly upregulated AKT, AMPK, and CPT-1, comparable to the effects of AT, PC, and ZO at 50 µg/mL (Figure 7B).

### 3.9. AT, PC, and ZO Regulated the Expression of Genes Related to Lipogenesis and Reduced FFA-Induced Intracellular Lipid Accumulation

**Figure 8:**
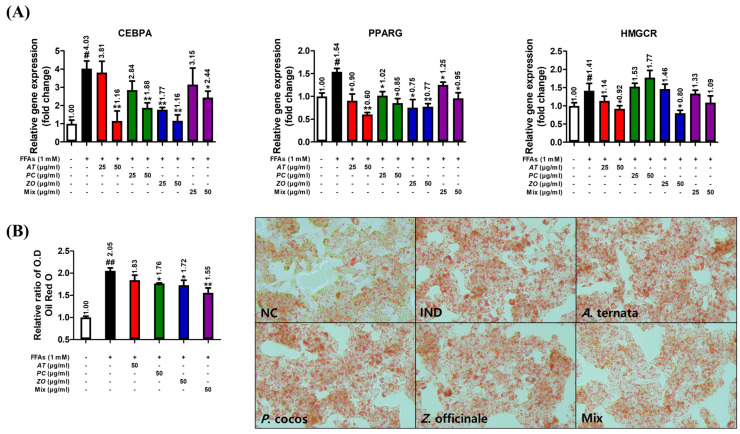
AT, PC, ZO, and MIX ameliorate lipogenesis in FFA-induced hepatic steatosis HepG2 cells. HepG2 cells were incubated in the absence or presence of FFAs (1 mM) with AT, PC, ZO, and MIX for six or 48 h. (**A**) Relative expressions of CEBPA, PPARγ, ACC, and HMGCR genes were determined by qPCR. (**B**) The impact on lipid accumulation was evaluated by measuring Oil Red O staining and comparing the microscopic images. Representative images (100×) of lipid accumulation via Oil Red O staining in HepG2 cells under different conditions. The results are presented as the means ± SDs of three independent experiments. # *p* < 0.05, ## *p* < 0.01 versus the FFA-treated controls, and * *p* < 0.05, ** *p* < 0.01 versus FFA-treated HepG2 cells.

The influence of herbal extracts on the gene expressions of CEBPA, PPARG, and HMGCR was investigated in FFA-induced HepG2 cells incubated with various concentrations of AT, PC, ZO, and MIX for 6 h or 48 h. HepG2 cells stimulated with 1 mM FFAs showed increased CEBPA, PPARG, and HMGCR mRNA expressions. AT and ZO prevented these upregulations. The mixture of AT, PC, and ZO regulated these, excluding the mRNA expression of HMGCR (Figure 8A). Furthermore, Oil Red O staining demonstrated the superior efficacy of MIX in reducing lipid accumulation both in optical density and in the microscopic analysis (Figure 8B).

## 4. Discussion

The holistic effects of herbal medicines were difficult to analyze because of their complex multi-components in extracts [54]. As therapeutic efficacy is based on the combined effects of mixed compounds, it was necessary to understand the complex interaction between multi-compounds from the herbal medicines and multi-targets of its compounds [55]. On the other hand, drug discovery is challenging and time-consuming when using conventional experiment-based screening methodologies [56].

In recent studies of oriental medicine, however, the network pharmacological approach allowed an understanding of the complex mechanisms of activity exerted by multiple compounds and an ability to predict the pharmacological activity [57,58]. This study deduced the targets, estimated to have a significant impact on hyperlipidemia based on the in silico study using network pharmacology analysis, and validated its real impact by performing an in vitro study. Several studies investigated the impact of natural products on hyperlipidemia using network pharmacology [15,59,60], but studies investigating herbal prescriptions (or their combinations) are relatively rare. In the present study, herbal combination exerted stronger activity than single herbs on hyperlipidemia and their related metabolic disorders, at least in certain aspects. Therefore, this result will arouse the interest of fellow researchers to conduct investigations on other herbal prescriptions on hyperlipidemia. 

Three major herbs with potential use in hyperlipidemia and its backgrounding metabolic disorders were deduced by analyzing the composition of the prescription based on the empirical knowledge databases of oriental medicine. Network pharmacology analysis revealed the key compounds from the herb combination that can modulate multiple targets critical to certain phenotype differentiation in oriental medicine for hyperlipidemia. In detail, a seemingly complex target network (PPI network) was divided into two different functional clusters of targets (Figure. 2). Many common genes, which are closely related to the pathologic mechanisms of hyperlipidemia were shared by two KEGG pathways ‘regulation of lipolysis in adipocytes’ and ‘PPAR signaling pathway’, as investigated by ClueGO.

The lipolysis process can be defined as the hydrolysis of triacylglycerol into fatty acids and glycerol to be used as an energy source [61]. This result might explain the observation of decreased neural lipid accumulation by three herbal extracts in the hepatic steatosis model, as demonstrated by the Oil Red O staining assay (Figure 7B).

The PPAR signaling pathway plays a key role in treating dyslipidemia by modulating the lipoprotein metabolism, which is targeted by fibrates [62,63]. Furthermore, the KEGG enrichment analysis of the functional clusters demonstrated its relationship with cholesterol metabolism and non-alcoholic fatty liver disease (Figure 4B,D). The enriched BP term results of these clusters are limited to cholesterol-related terms, as well as triglycerides, sterol, and lipid homeostasis (Figure 4A). This appears to be relative merit of the herbal combination to current statin treatment, of which its primary drug mechanism is to inhibit the rate-limiting enzyme for cholesterol synthesis [64]. In addition, five significantly enriched KEGG pathways (*p* < 0.05), which are strongly involved in lipid metabolism within the rank of the top 20, were found (Table 2).

The combinational effect was evaluated by comparing the efficacy of single extracts and mixed extracts. As shown in the in vitro data, a mixed extract of three herbs and single-herb extracts against FFA-induced hepatic steatosis had a notable effect on the lipid biosynthesis genes (Figure 7). Moreover, as a complex mixture of bioactive compounds, it has several additional benefits on energy expenditure (by phosphorylating AMPK) or lipid catabolism (by increasing CPT1a expression) in the aspects of treating dyslipidemia-related complications (Figure 6). On these markers, even more favorably, the mixed extract exerted an enhanced efficacy compared to single-herb extracts. This is more advantageous when patients with hyperlipidemia usually have comorbidities of other metabolic disorders related to obesity [65,66]. In addition, the STITCH result suggested that the targets of the herbal combination have a close interaction with several statins that might imply a similar drug mechanism. Therefore, the herbal mixture might provide an option to treat hyperlipidemia with new molecular targets and mechanisms that can ameliorate metabolic disorders and ultimately alternate statins. 

However, this study presented limited evidence of the possibility of the herbal combination on hyperlipidemia and other metabolic disorders. The 41 key target genes do not include the core targets related to the lipid metabolism, such as SREBF1 and SCD1 (Table 2). Moreover, not all the targets could be validated in the experiments deduced from network pharmacology. The herbal combination sample might show efficacy in the steatosis model but not in hyperlipidemia because it could not be determined that the reduced accumulation of lipid droplets was not caused by decreased fatty acid uptake in media.

The hypolipidemic efficacy of the herbal combination needs to be validated in in vivo studies. In particular, a comparative study to evaluate the overall efficacy of the herbal combination on metabolic disorders, not only for dyslipidemia, should be conducted in comparison with the current statin doses.

## 5. Conclusions

The pharmacological activities of three herbs, including ZO, AT, PC against dyslipidemia, were estimated via network pharmacological approaches. The herbs potentially target several key proteins and pathways critical to lipid metabolism. The efficacy and mechanism of the herbal extracts were investigated in hepatic steatosis model in vitro. The mixed herb extract showed a stronger potential against hepatic steatosis compared to single-herb extracts. As a result, we suggest that the herbal combination could be a candidate drug for hyperlipidemia which can alternate statin, with significant benefits on modulating lipid metabolism.

## Figures and Tables

**Table 1:** Compounds of *Zingiber officinale*, *Poria cocos* and *Arum ternata*.

Compound List				
Herb	Name	CID	OB	DL
*Zingiber officinale* (ZO)	(10)-Gingerol	168115	19.14	0.28
	10-Gingerdione	5317591	21.42	0.29
	6-methylgingediacetate2	53179662	48.73	0.32
	shogaol	5281794	31.00	0.14
	poriferast-5-en-3beta-ol	457801	36.91	0.75
	thymol	6989	41.47	0.03
*Poria cocos* (PC)	Cerevisterol	10181133	37.96	0.77
	Dehydroabietic acid	94391	14.93	0.28
	(2R)-2-[(3S,5R,10S,13R,14R,16R,17R)-3,16-dihydroxy-4,4,10,13,14-pentamethyl-2,3,5,6,12,15,16,17-octahydro-1H-cyclopenta[a]phenanthren-17-yl]-6-methylhept-5-enoic acid	10743008	30.93	0.81
	Ergosterol peroxide	5351516	40.36	0.81
	ergosta-7,22E-dien-3beta-ol	5283628	43.51	0.72
	hederagenin	73299	22.42	0.74
	trametenolic acid	12309443	38.71	0.80
*Arum ternata* (AT)	(3S,6S)-3-(benzyl)-6-(4-hydroxybenzyl)piperazine-2,5-quinone	11438306	46.89	0.27
	10,13-eicosadienoic	549062	39.99	0.20
	3,4-Dihydroxybenzaldehyde	8768	38.35	0.03
	4-Methoxybenzoic acid	7478	29.69	0.03
	8-Octadecenoic acid	5282758	33.13	0.14
	Baicalein	5281605	33.52	0.21
	Xanthosine	64959	44.72	0.21
	caffeic acid	689043	25.76	0.05
	oct-1-ene	8125	39.25	0.01
	Baicalin	64982	40.12	0.75
	Choline	305	0.47	0.01
	9-oxononanoic acid	75704	19.60	0.03
	docosanoic acid	8125	15.69	0.26
	9-Heptadecanol	136435	14.24	0.09
	Palmitic acid	985	19.30	0.10
	Baicalein	5281605	33.52	0.21
	Hydroquinone	785	29.26	0.02
	Anethole	637563	32.49	0.03
	Adenine	190	62.81	0.03
	Cavidine	193148	35.64	0.81
	Chrysophanol	10208	18.64	0.21
	Coniferin	5280372	10.28	0.27
	Cycloartenol	92110	38.69	0.78
	Ephedrine	9294	43.35	0.03
	Furfural	7362	34.35	0.01
	gondoic acid	5282768	30.70	0.20
	Homogentisic acid	780	92.44	0.04
	Linoleic acid	5280450	41.90	0.14
	Oleic acid	445639	33.13	0.14
	Pentadecanoic acid	13849	20.18	0.08
	Sitogluside	5742590	20.63	0.62
	Stigmast-4-en-3-one	5484202	36.08	0.76
	Thymidine	5789	11.34	0.11
AT ∩ ZO	beta-sitosterol	222284	15.00	0.81
	Stigmasterol	5280794	43.83	0.76
AT ∩ ZO ∩ PC	Palmitic acid	985	19.30	0.10

**Table 2:** Network parameters analyzed from the total PPI network.

	Degree	Stress	Betweenness Centrality
AKT1	31	894	0.131579
PPARG	27	682	0.092949
PTGS2	26	494	0.049889
CAT	26	484	0.049086
VEGFA	25	454	0.042859
PPARA	23	448	0.049087
CRP	23	542	0.066034
IL10	22	238	0.018832
SERPINE1	21	476	0.049813
MMP9	19	164	0.011888
HIF1A	19	178	0.012385
PLG	18	472	0.057540
ESR1	17	154	0.010555
PTEN	16	228	0.023134
MPO	15	314	0.053684
TGFB1	14	26	0.001131
SELP	12	98	0.007016
FASN	12	104	0.011726
PON1	11	100	0.012281
LPL	11	72	0.006200
CD40LG	11	42	0.004880
AR	11	94	0.010259
GCG	11	42	0.005491
AHR	11	30	0.001772
SOD1	10	12	0.000844
HMGCR	9	256	0.051766
DDIT3	9	26	0.001189
CYP1A1	9	50	0.005523
BAX	8	8	0.000513
PTGS1	8	4	0.000256
AKR1B1	7	2	0.000056
FABP1	7	18	0.001893
CETP	6	6	0.000569
F7	5	32	0.005104
PIK3CG	4	2	0.000214
ACHE	4	6	0.000341
ADRB2	3	0	0.000000
NR3C2	3	8	0.000383
SERPIND1	2	0	0.000000
LYZ	1	0	0.000000
SOAT1	1	0	0.000000

**Table 3:** Enriched KEGG pathway obtained from DAVID database.

	KEGG Pathway		
Entry	Pathway	FDR	Genes
hsa04923	Regulation of lipolysis in adipocytes	0.00039	AKT1, PTGS1, PTGS2, ADRB2
hsa04932	Non-alcoholic fatty liver disease	0.00054	AKT1, BAX, DDIT3, PPARA, TGFB1
hsa03320	PPAR signaling pathway	0.00062	FABP1, LPL, PPARA, PPARG
hsa04152	AMPK signaling pathway	0.0019	AKT1, FASN, HMGCR, PPARG
hsa04979	Cholesterol metabolism	0.0023	CETP, LPL, SOAT1

## Data Availability

Not applicable.
